# Time and spatial trends in landing per unit of effort as support to fisheries management in a multi-gear coastal fishery

**DOI:** 10.1371/journal.pone.0258630

**Published:** 2022-07-01

**Authors:** Pedro Leitão, Luis Sousa, Margarida Castro, Aida Campos

**Affiliations:** 1 IPMA, Instituto Português do Mar e da Atmosfera, Algés, Portugal; 2 CCMAR, Centro de Ciências do Mar, Universidade do Algarve, Faro, Portugal; MARE – Marine and Environmental Sciences Centre, PORTUGAL

## Abstract

Landings by the multi-gear coastal fleet operating off the Portuguese continental coast include about 300 species, from which only a few are the object of management plans. In this study, daily landings (kg trip^-1^) are used, along with an effort indicator, vessel length overall (LoA), to obtain landings per unit of effort (LPUE) as a proxy for the species relative abundance, for a total of 48 species. LPUE indices were used as a response variable in linear models where year (2012–2016), season, region (north and south) and NAO index were explanatory variables. Seasonal and regional effects were found to significantly affect species abundance for a total of 41 and 40 species respectively, while year trends were found to be significant for 19 species, and the NAO index for 3 species. LPUE density maps are presented for several selected species and a subsample of trips, where VMS records were available. It is proposed that geographic and seasonal changes in LPUE can be used to understand trends in abundance and obtain information that can be used in support regional management plans.

## Introduction

In the European Union (EU), the principles underlying the Common Fisheries Policy (CFP) focus on long-term sustainability of marine resources. Fisheries were recognized to be dependent on healthy marine ecosystems, requiring the integration of the fisheries sector in other policies dealing with marine activities, namely the Integrated Maritime Policy (IMP) and its environmental pillar, the Marine Strategy Framework Directive—MSFD (Directive 2008/56/EC of the European Parliament and of the Council of 17 June 2008) establishing a framework for community action in the field of marine environmental policy. An important aspect of these policies is the implementation of the ecosystem-based approach to fisheries management (EAFM) which became a central issue under the CFP, requiring information on the state of the marine environment including indicators of fishing pressure, both for target and non-target species [1, 2]. The implementation of an EAFM system also implies a regional approach to fisheries management, increasing stakeholder participation with the establishment of fishery-based management plans, and mitigation measures to be tailored to specific fisheries [3]. These will ultimately result in multi-species long-term plans considering the spatial component of each fishery.

The EAFM calls upon a fleet- and area-based approach to fisheries management (Council Regulation (EC) No 1343/2007) increasingly requiring control of the catch composition with limited access to given areas, reducing ecosystem impacts resulting from fishing activities. This has placed input control of catches, including fishing effort limitations and technical conservation measures (gear restrictions, species minimum conservation reference sizes (MCRS) and real time closures), as key management measures along with output controls (e.g., total allowable captures (TACs) and quotas). Today, a combination of both types of measures is used in European fishery policies [4]. Thus, fisheries dependent data, i.e., data collected at the scope of the fishing activity including reported catches, landings and georeferenced data, are gaining increasing importance in fisheries management.

The evolution of methods for the assessment and management of fishing resources took place in parallel with important changes in data collection [5]. Currently, most of the information relevant to fisheries management in the EU is obtained either through vessel research surveys or fleet monitoring programs under the Data Collection Framework (DCF) [5]. In the last two decades, new mandatory procedures evolved to improve and standardize fisheries data throughout the EU [6]. Large and more reliable datasets are generated, and statistical modelling can be applied to retrieve crucial fisheries information such as the identification of exploited species and their distribution [7–9]. The International Council for the Exploration of the Sea (ICES), the entity responsible for providing advice to the EU on fisheries issues, has identified, as a major objective in its Science Plan, the development of effective mechanisms to use monitoring and surveillance data to support scientific advice [10].

Traditional stock assessment has relied on fisheries independent surveys (scientific campaigns) to evaluate the biomass of exploited resources [11, 12]. In the absence of this information, fisheries dependent data, such as catch per unit of effort (CPUE), represents the best available indicator of abundance [13–15].

Fisheries dependent data is particularly useful for the management of multi-species fisheries when only a few are subject to formal assessment, resulting in TACs and quotas. For these fisheries, alternative management methods should be developed, including area-specific management based on easily accessible fisheries dependent data. This approach assumes particular importance for fleets not covered by onboard sampling programs [16, 17].

For data deficient fleets, landings, instead of catches, can be used to obtain an alternative abundance index, landings per unit of effort (LPUE) [18, 19]. Landings, combined with georeferenced data on the fishing activity, can provide information on stock trends at a regional level, leading to better conservation and advice [20]. Near real-time closures (RTCs) based on daily high-definition fishing maps of CPUE are beneficial to fisheries, in particular for heavily fished demersal species [21, 22]. For example, Iceland and Scotland rely on georeferenced catch data to define RTCs, limiting fishing in particular areas and ensuring sustainability [21].

The multi-gear fishing fleet operating on the Portuguese continental coast (ICES Division IXa) accounts for 96% of the total number of vessels, employs 69% of fishers and is responsible for 31% of the landings in weight and 59% in value [23]. This complex fleet operates year-round, over a great variety of ecosystems, adapted to regional and seasonal availability of resources. Most vessels are licensed for multiple fixed fishing gears (such as gillnets and trammel nets, longlines, traps and pots) to capture a great diversity of benthic, demersal and pelagic species (fish, shellfish, cephalopods, and crustaceans). Due to safety regulations and habitability conditions of fishing vessels, only a small fraction of the multi-gear fleet can accommodate onboard observers to gather information for the EU Data Collection Framework program (DCF) [5].

The objective of this study is to illustrate the use of daily landing records and effort to obtain LPUE indexes as a proxy of abundance for several species captured by a multi-gear, multi-species fleet, and then relate the LPUE indexes with spatiotemporal indexes. For a selection of species with available georeferenced information given by VMS, maps of LPUE were produced to illustrate spatiotemporal exploitation patterns. This information can contribute to define regional management plans such as, for example, closed areas, closed seasons or catch/landing restrictions.

## Materials and methods

### Data sources

The Portuguese multi-gear fleet, operating mainly nets, traps and longlines, account for relatively low bycatch when compared to other fishing activities such as bottom trawling. It generally takes an opportunistic “all species are valuable” approach, discarding only species interdicted or those with no commercial value [24–26]. In such conditions, landings and catches are closely related and, in the absence of catch per unit of effort (CPUE), landings per unit of effort (LPUE) are considered as a good proxy for species abundance [19].

The data analysis refers to the multi-gear coastal fleet operating off the continental Portuguese coast, comprising a total of 492 vessels greater than 9 meters in length. Although most vessels use different types of static gear, a small fleet sub-segment, of approximately 40 vessels used in this analysis, operates only dredges to exclusively target bivalves. For each fishing trip, the data consisted of daily landings by species and technical characteristics of the vessels. For a sample of 165 vessels greater than 15 meters, VMS records were also available.

#### Landings data

Daily landings information for the 492 vessels belonging to the multi-gear coastal fleet included: date, landing port, vessel ID, species identification (common name and the 3-alpha FAO code) and both landings weight (kg) and value (€ kg^-1^). The original landings dataset comprised a total of 257,134 fishing trips and 297 species. Three criteria were applied in sequence, to eliminate very rare species and/or trips with very low catches: (1) selection of the 100 species with highest landing frequencies, (2) selection of the 50 species with highest total landings (in weight), (3) for each of these 50 species, selection of the trips that landed more than 10% of the average daily landing of that species (e.g., if the average of a particular species for day X is 10kg, then trips with less than 1kg in day X were excluded for the calculation of LPUE). Later, the genus *Microchirus* was removed due to duplicated records as genus and species. The final dataset included 48 species captured in 247,252 trips, representing a loss of 3.8% of the original number of trips.

#### Vessel characteristics and VMS data

The technical characteristics of the vessels comprised length overall (LoA), gross tonnage (GT), vessel power in kilowatts, year of construction and port of register.

Regarding VMS, the coastal fleet of interest is composed of vessels with length overall (LoA) greater than 9 meters, but VMS systems are only installed in some vessels larger than 12 meters and in all vessels larger than 15 meters. Vessels between 12 and 15 meters are exempt from having the monitoring equipment if they operate exclusively within territorial waters or spend less than 24 hours at sea. Due to this, of the 492 vessels in the multi-gear coastal fleet, only 165 were equipped with VMS, representing approximately 90,000 trips. VMS positioning data for each vessel consists of a succession of geographical locations (latitude, longitude), timestamp, speed, and course, received by a ‘blue-box’ (satellite-tracking device installed on board the fishing vessels). This information is transmitted via satellite to the Fisheries Control Centre every 2 hours.

At an initial data processing stage, VMS data were filtered to exclude records with duplicate or erroneous values. The data analysis proceeded with the identification of fishing trips (FT) for the 165 vessels. This identification was carried out by partitioning the VMS data into sections starting with departure from a port and ending at the arrival to the same or a different port. Each one of these sections, corresponding to a fishing trip, was associated to a landing declaration. The objective was, for each species, to provide spatial-temporal information on fishing grounds and relative abundance, together with information on fishing effort. The VMS data, landings and vessel technical characteristics were provided in an anonymized format (each vessel was attributed a code) by the Directorate-General for Natural Resources, Safety and Maritime Services (DGRM).

#### Geographical, temporal, and environmental variables

Previous studies on spatial distribution of fish assemblages off the Portuguese continental shelf, based on the analysis of trawl surveys [27], demonstrated the existence of two main biological regions, separated by a steep-side valley, the Nazaré Canyon. This feature represents not only a physical obstacle for fish communities but is also a boundary between two areas that are geologically and environmentally different. The southern continental shelf is narrow and affected by weak outward winds, while the northern shelf is wider and is influenced by southward coastal winds, creating the conditions for upwelling and higher primary production and leading to higher pelagic fish abundance [28, 29]. This boundary was considered in the present study, using the landing port to associate the fishing activity to a region, north or south, in relation to the Nazaré canyon.

Two variables related to time were considered as possibly influencing LPUE: year (2012 to 2016) and season (winter from January to March, spring from April to June, summer from July to September and fall from October to December).

An environmental variable was included, the North Atlantic Oscillation (NAO) index. This index is related to the difference between low atmospheric pressure at high latitudes and high atmospheric pressure at low latitudes in the North Atlantic. Its magnitude depends on the choice of the sampling sites where high and low atmospheric pressures are measured, as well as on the seasons chosen [30]. In this work we used the NAO index proposed by [31] that considers the difference of normalized sea level pressure between the Azores (high) and Iceland (low). The NAO influences the direction and strength of western winds, and high index values during the previous winter may have a positive effect on primary production [30, 32], positively affecting zooplankton abundance and favouring larval survival and fish recruitment [33, 34].

For each species, a lag (δ in years) was obtained, representing the age of minimum conservation reference size (MCRS, the minimum size reported in commercial fisheries). Thus, the length at MCRS was converted into age at MCRS, based on available age-length relationships (values for the Portuguese coast were used, when not available the closest area was considered). It was assumed that age at MCRS is a good indicator of age of recruitment to the fishery and thus LPUE of a given year Y was associated with the NAO index of year Y-δ (table with lag values and references in S1 Table).

#### Standardized fishing effort and LPUE

Detailed information on fishing effort was not available for the fleet of interest. However, Portuguese fishing regulations, Ordinance n° 1102-H/2000, defines six vessel size categories, with corresponding maximum gear length. Due to this, vessels with higher LoA are allowed to use two to three times more gears, resulting in higher fishing effort. Thus, vessel length was considered a proxy for fishing capacity. A second indicator is the engine power, commonly used to standardize fishing effort in trawlers [5]. These two potential indicators of the vessels’ fishing capacity (length overall and engine power) were investigated through their relationship with landed weight. Although both variables were significant (p-values < 0.001), a smaller p-value was present between LoA and landed weight, so this variable was chosen to indicate fishing effort.

Daily landings per unit of effort (LPUE_*s*,*d*_) for each species was calculated through Eq 1:

$${LPUE}_{s,d}=\frac{\sum^{v}W_{s,d,v}}{\sum^{v}{LoA}_{s,d,v}}$$ Eq 1

where the landed weight (*W; kilograms*) for species *s*, day *d* and vessel *v*, is summed for all selected vessels (more than 10% criterion referred above) and divided by the sum of the vessel LoA (meters).

### Statistical methods

General linear models were used in this study to evaluate the influence of multiple variables on LPUE. Those relationships have been used to detect significant associations that can be meaningful to explain variations in LPUE. The variables used in the statistical analysis were LPUE per day and species as response variable (log transformed), year and NAO index as continuous explanatory variables and region and season as categorical explanatory variables. The model, adjusted for each of the 48 species selected, was:

$$Log_{10}(LPUE)=\beta_{0}+\beta_{Y}Y+\beta_{S}S+\beta_{x}X+\beta_{R}R+\varepsilon$$ Eq 2

where LPUE is landings per unit of effort (kg per metre boat length per day), Y = year, S = season, X = NAO index, R = region and ε = error.

The p-values of the specific terms of the model, with a Bonferroni correction for multiple tests, were used to assess statistical significance and strength of the explanatory variables [35]. Significance was discussed only for more important variables with p-values less than 0.01.

Database setup and statistical analysis were carried out using R version 3.6.3 on RStudio.

### Mapping LPUE

Maps of relative abundance were obtained for eight selected species, using QGIS version 3.10. Daily landings of a particular species and vessel were assigned a trip trajectory by dividing the landed weight for that species by the number of corresponding VMS records of that trip. In this process, only VMS records with speeds equal or below 3.5 knots were considered, assumed to be unequivocally associated to gear haul-up. This threshold was based on the analysis of the frequency distribution of vessels speed records and further validated through interviews to skippers involved in the coastal multi-gear fleet.

Global LPUE heatmaps were thus created for each species. A global map of all VMS points used (all species) was also produced to indicate the area covered in this study.

## Results

The three most important species for the fleet analysed and the period considered, both in quantity and landed value are the common octopus (*Octopus vulgaris*), the black scabbardfish (*Aphanopus carbo*) and the European hake (*Merluccius merluccius*), representing 50% of the landings. However, while the two former species are almost exclusively landed by the multi-gear fleet, European hake is mostly landed by coastal trawlers. Landings for multi-gear fleet represent only 40% of the landed weight for this species and, furthermore, European hake is subject to formal assessment. Therefore, it will not be analysed here. The next group of species, by order of importance of the landings in weight, are Atlantic horse mackerel (*Trachurus trachurus*), pouting (*Trisopterus luscus*), surf clam (*Spisula solida*), European conger (*Conger conger*), swordfish (*Xiphias gladius*), thornback ray (*Raja clavata*) and blue shark (*Prionace glauca*), altogether comprising around 27% of the total landings. In value, again by decreasing order of importance, the species ranking four to ten and representing 25% of the revenue are swordfish, common sole (*Solea solea*), surf clam, John dory (*Zeus faber*), pouting, angler (*Lophius piscatorius*) and European conger. Data with total landings in weight and value, ranks and relative importance are presented for each species as S2 Table.

The five species with highest median LPUE (more than 20 kg LoA^-1^ trip^-1^) are, by decreasing order, black scabbardfish, surf clam, swordfish, Atlantic pomfret (*Brama brama*) and smooth clam (*Callista chione*) (data for all species presented as S3 Table).

**Fig 1:**
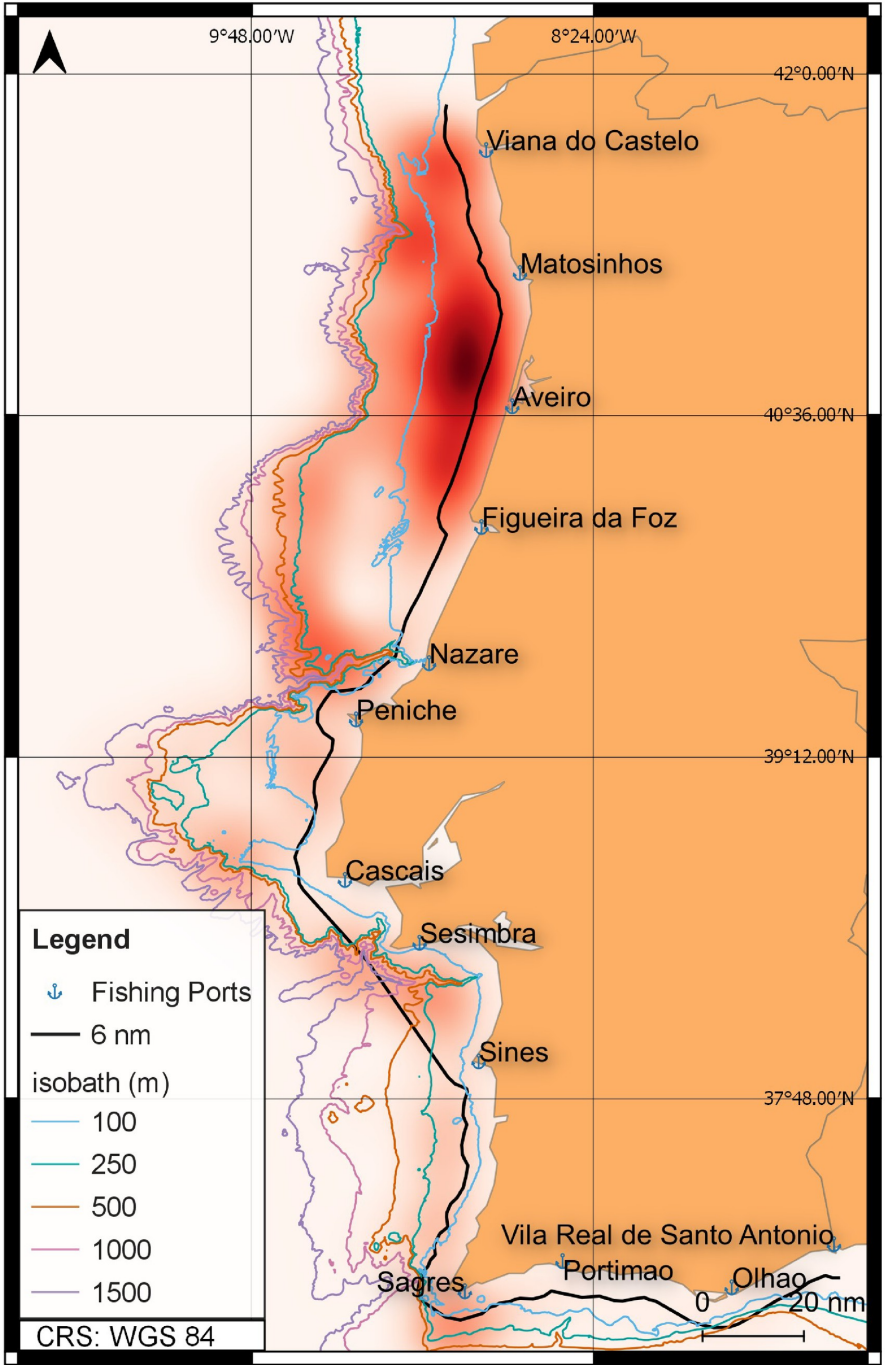
Spatial distribution of the fishing intensity for the multi-gear coastal vessels with available VMS records from 2012 to 2016 in mainland Portugal. Main fishing ports are indicated as well as the 6 nautical miles line corresponding to the inner limit of trawling activity, while the multi-gear fleet operates mostly between the 6 nm and the coast. Only points corresponding to speeds below 3,5 knots (kt) were considered in the heatmap, assumed to be associated with fishing activity. Bathymetry from the EMODnet Bathymetry Consortium [36].

The spatial distribution of the fishing activity for vessels where VMS data was available is represented in Fig 1 (the red area is a heatmap of all the VMS points identified as fishing activity).

The fishing activity extends along the entire coast until the 500 metres isobath, with higher intensity to the north of the Nazaré canyon, where the continental shelf is more extended. Between the Nazaré and Setúbal canyons, the bottom is characterized by numerous physiographic features (the Central Portuguese submarine canyons, Nazaré, Cascais and Setúbal–Lisbon canyons, [37]). In these regions, there are multiple rocky areas unsuitable for trawling, opening the opportunity for fixed gears inside the 6 nm. South of Sesimbra and along the south coast, most of the multi-gear fleet activity is inside the 250 m isobath. This is related to the intense exploitation of the continental slope by trawlers targeting mostly deep-water crustaceans. All along the coast, narrow strips following the 1000 m isobath (north of Sesimbra) and the 1500 m isobath (between Sesimbra and Sagres) and along canyons, constitute the fishing grounds for longline fisheries targeting mostly black scabbardfish.

The results of the linear model applied to the variables year, NAO index, season and region are presented in Table 1, for each of the 48 species considered, including the coefficients for the continuous variables (year and NAO index) and the indication of the level of each factor (season and region) with higher LPUE. Overall, the two explanatory variables that showed a higher number of significant correlations were season (41 species) and region (40 species). Three bivalve species were only present in the southern region, making region relevant for 44 of the 48 species considered.

**Table 1:** Results of linear model for each of the 48 species considered.

Common name	Scientific name	FAO code	Year coef.	Year p-value	Season	Season p-value	NAO coef.	NAO p-value	Region	Region p-value
*Actinopterygii*										
Angler	*Lophius piscatorius*	MON	**-0.06**	**<0.0001**	**SP**	**<0.0001**		~1.0000	**S**	**<0.0001**
Atlantic horse mackerel	*Trachurus trachurus*	HOM		~1.0000	**WI**	**<0.0001**		~1.0000	**N**	**<0.0001**
Atlantic mackerel	*Scomber scombrus*	MAC		0.1784		~1.0000		~1.0000	**N**	**<0.0001**
Atlantic pomfret	*Brama brama*	POA	**-0.54**	**<0.0001**	**WI**	**<0.0001**		~1.0000	**S**	**0.0001**
Axillary seabream	*Pagellus acarne*	SBA	**-0.03**	**<0.0001**	**FA**	**<0.0001**		0.0180	**S**	**<0.0001**
Black scabbardfish	*Aphanopus carbo*	BSF		~1.0000	**WI**	**<0.0001**		~1.0000	**S**	**<0.0001**
Black seabream	*Spondyliosoma cantharus*	BRB		~1.0000		0.7125		~1.0000	**S**	**<0.0001**
Blackbellied angler	*Lophius budegassa*	ANK	**-0.05**	**<0.0001**	**FA**	**<0.0001**		~1.0000	**S**	**<0.0001**
Blackbelly rosefish	*Helicolenus dactylopterus*	BRF		~1.0000	**SP**	**<0.0001**		~1.0000	**S**	**<0.0001**
Blackspot seabream	*Pagellus bogaraveo*	SBR		0.0283	**WI**	**<0.0001**		~1.0000	**S**	**<0.0001**
Chub mackerel	*Scomber japonicus*	MAS	**-0.09**	**<0.0001**	**SU**	**<0.0001**		~1.0000	**N**	**0.0001**
Common sole	*Solea solea*	SOL		0.1063	**WI**	**<0.0001**		0.1407	**N**	**<0.0001**
Common two-banded seabream	*Diplodus vulgaris*	CTB		~1.0000	**FA**	**<0.0001**		~1.0000	**S**	**<0.0001**
European conger	*Conger conger*	COE		~1.0000	**SU**	**<0.0001**		~1.0000	**S**	**<0.0001**
European hake	*Merluccius merluccius*	HKE	**-0.02**	**0.0075**	**SU**	**<0.0001**	**-0.03**	**0.0041**		~1.0000
European seabass	*Dicentrarchus labrax*	BSS		~1.0000	**WI**	**<0.0001**		0.5336	**S**	**<0.0001**
Forkbeard	*Phycis phycis*	FOR	**-0.04**	**<0.0001**	**SP**	**<0.0001**		0.2264	**S**	**<0.0001**
John dory	*Zeus faber*	JOD	**-0.04**	**<0.0001**	**SU**	**<0.0001**		0.4816	**S**	**<0.0001**
Large-scaled gurnard	*Lepidotrigla cavillone*	LDV		0.7428	**WI**	**<0.0001**		~1.0000	**N**	**<0.0001**
Meagre	*Argyrosomus regius*	MGR		~1.0000	**FA**	**<0.0001**		0.8065	**S**	**<0.0001**
Pouting	*Trisopterus luscus*	BIB		0.1422	**SU**	**<0.0001**		~1.0000	**N**	**<0.0001**
Red gurnard	*Aspitrigla cuculus*	GUR		1.0000	**WI**	**<0.0001**		~1.0000	**S**	**<0.0001**
Red porgy	*Pagrus pagrus*	RPG		0.6288	**FA**	**<0.0001**		~1.0000	**S**	**<0.0001**
Sand sole	*Pegusa lascaris*	SOS	**-0.06**	**<0.0001**	**SP**	**<0.0001**		~1.0000	**S**	**<0.0001**
Silver scabbardfish	*Lepidopus caudatus*	SFS	**0.09**	**0.0002**		~1.0000		~1.0000	**S**	**<0.0001**
Surmullet	*Mullus surmuletus*	MUR		0.7390	**FA**	**<0.0001**		~1.0000	**S**	**<0.0001**
Swordfish	*Xiphias gladius*	SWO		0.6247	**FA**	**<0.0001**		~1.0000	**S**	**<0.0001**
Tub gurnard	*Chelidonichthys lucerna*	GUU		0.8692	**WI**	**<0.0001**		~1.0000	**N**	**<0.0001**
Wedge sole	*Dicologlossa cuneata*	CET		~1.0000	**SU**	**<0.0001**		~1.0000	**N**	**<0.0001**
Whiting	*Merlangius merlangus*	WHG	**-0.06**	**<0.0001**	**FA**	**<0.0001**	**0.04**	**0.0025**	**N**	**<0.0001**
Wreckfish	*Polyprion americanus*	WRF	**-0.07**	**<0.0001**	**SU**	**<0.0001**		0.7327	**S**	**<0.0001**
*Chondrichthyes*										
Blonde ray	*Raja brachyura*	RJH	**-0.06**	**<0.0001**	**SU**	**<0.0001**		~1.0000	**S**	**<0.0001**
Blue shark	*Prionace glauca*	BSH		~1.0000	**SU**	**0.0047**		~1.0000	**S**	**<0.0001**
Lowfin gulper shark	*Centrophorus lusitanicus*	CPL		0.0901	**SU**	**<0.0001**		~1.0000	**N**	**<0.0001**
Nursehound	*Scyliorhinus stellaris*	SYT		~1.0000	**SP**	**<0.0001**		~1.0000	**S**	**<0.0001**
Shortfin mako	*Isurus oxyrinchus*	SMA	**-0.19**	**<0.0001**	**FA**	**<0.0001**		~1.0000	**S**	**<0.0001**
Smooth-hound	*Mustelus mustelus*	SMD		~1.0000		0.2634		0.0117	**S**	**<0.0001**
Spotted ray	*Raja montagui*	RJM	**-0.03**	**<0.0001**	**WI**	**<0.0001**		~1.0000		~1.0000
Thornback ray	*Raja clavata*	RJC		~1.0000	**SU**	**<0.0001**		~1.0000	**N**	**<0.0001**
Tope Shark	*Galeorhinus galeus*	GAG		~1.0000		~1.0000		~1.0000		0.2906
*Cephalopoda*										
Common octopus	*Octopus vulgaris*	OCC		0.9520	**FA**	**<0.0001**		~1.0000		0.3171
Cuttlefish	*Sepia officinalis*	CTC		~1.0000	**WI**	**<0.0001**	**0.07**	**<0.0001**	**S**	**<0.0001**
Neon flying squid	*Ommastrephes bartramii*	OFJ	**0.03**	**0.0032**	**SU**	**<0.0001**		~1.0000	**N**	**<0.0001**
*Bivalves*										
Bean clams	*Donax spp*	DON		0.0403		0.5642		~1.0000		
Pod razor	*Ensis siliqua*	EQI	**0.18**	**<0.0001**		0.1393		0.0310		
Smooth clam	*Callista chione*	KLK		~1.0000	**FA**	**<0.0001**		~1.0000		
Stripped Venus clam	*Chamelea gallina*	SVE		0.3698	**SP**	**<0.0001**		~1.0000		~1.0000
Surf clam	*Spisula solida*	ULO	**0.11**	**<0.0001**	**FA**	**<0.0001**		0.7979	**N**	**<0.0001**

The response variable is LPUE (landings-per-unit-effort). All p-values (Bonferroni adjusted) are reported. Significance was considered for p-values<0.01 (in bold). When significant, the coefficients for year and NAO index are presented. For season (FA–fall, SP–spring, WI–winter, SU–summer) and region (N–north, S–south), when significant, the factor level with higher LPUE is indicated. The species are sorted by alphabetic order of their common names inside each taxonomic class.

A total of 18 species presented yearly trends in the period in study, of which 4 showed a positive and 14 a negative trend. NAO index was significant for 3 species: European hake, whiting (*Merlangius merlangus*) and cuttlefish (*Sepia officinalis*).

A total of 31 species were analysed in the Actinopterygii class. Except for the European hake, all species presented regional differences with respect to LPUE. Nine species had significantly higher LPUE in the north (Atlantic horse mackerel, Atlantic mackerel (*Scomber scombrus*), chub mackerel (*Scomber japonicus*), common sole, large-scaled gurnard (*Lepidotrigla cavillone*), pouting, tub gurnard (*Chelidonichthys lucerna*), wedge sole (*Dicologlossa cuneata*) and whiting), while for the remaining species LPUE was significantly higher in the south. Twelve species displayed significant interannual variations, 11 of which with negative and one with positive trends. The former group comprised angler, Atlantic pomfret, axillary bream (*Pagellus acarne*), blackbellied angler (*Lophius budegassa*), chub mackerel, European hake, forkbeard (*Phycis phycis*), John Dory, sand sole (*Pegusa lascaris*), whiting and wreckfish (*Polyprion americanus*). The silver scabbardfish (*Lepidopus caudatus*) was the only species presenting a positive trend. Seasonal variations were significant for a total of 28 species, with higher LPUEs during the winter for nine (Atlantic horse mackerel, Atlantic pomfret, black scabbardfish, blackspot seabream (*Pagellus bogaraveo*), common sole, European seabass, large-scaled gurnard, red gurnard (*Aspitrigla cuculus*) and tub gurnard), during the spring for four species (angler, blackbelly rosefish (*Helicolenus dactylopterus*), forkbeard and sand sole), during the summer for seven species (chub mackerel, European conger, European hake, John Dory, pouting, wedge sole and wreckfish) and during the fall for the remaining eight species (axillary seabream, blackbellied angler, common two-banded seabream (*Diplodus vulgaris*), meagre (*Argyrosomus regius*), red porgy (*Pagrus pagrus*), surmullet (*Mullus surmuletus*), swordfish and whiting). The NAO index was significantly different for two *Actinopterygii* species LPUEs, with higher LPUEs for negative NAO indexes for European hake and positive indexes towards whiting.

In the Chondrichthyes class, season was significant for seven out of the nine species analysed: blonde ray (*Raja brachyura*), blue shark, lowfin gulper shark, thornback ray (LPUE significantly higher in the summer), nursehound (*Scyliorhinus stellaris*; in the spring), shortfin mako (*Isurus oxyrinchus*; in the fall) and spotted ray (*Raja montagui*; in the winter). Geographic differences were detected for seven species, two with higher LPUE in the north, lowfin gulper and thornback ray, while the remaining five species, blonde ray, blue shark, nursehound, shortfin mako and smooth-hound (*Mustelus mustelus*), with higher values in the south. A negative trend over time was significant for three species, shortfin mako, blonde ray, and spotted ray. One species, the tope shark (*Galeorhinus galeus*) did not show significant association with any of the explanatory variables in the model. Season was significant for the three cephalopod species, the common octopus, the cuttlefish, and the neon flying squid (*Ommastrephes bartramii*), with higher LPUE in the fall, winter and summer, respectively. Regional differences were significant for cuttlefish and neon flying squid, the first was more abundant in the north and the later in the south. Year was significant with a negative trend for the neon flying squid. For cuttlefish, the correlation between LPUE and the NAO index was significant and positive.

Of the five bivalve species, bean clam (*Donax spp*), pod razor (*Ensis siliqua*) and smooth clams were exclusively landed in the south, while regional differences were detected for surf clam, with higher LPUEs in the north. Pod razor and surf clam presented an increasing trend with respect to the year, while seasonal patterns were present for the smooth and surf clams, with higher LPUE in the fall, and for the stripped Venus clam (*Chamelea gallina*), with higher LPUE in the spring. The bean clam did not show any significant correlation, although region is implicitly important since this species was caught only in the south.

**Fig 2:**
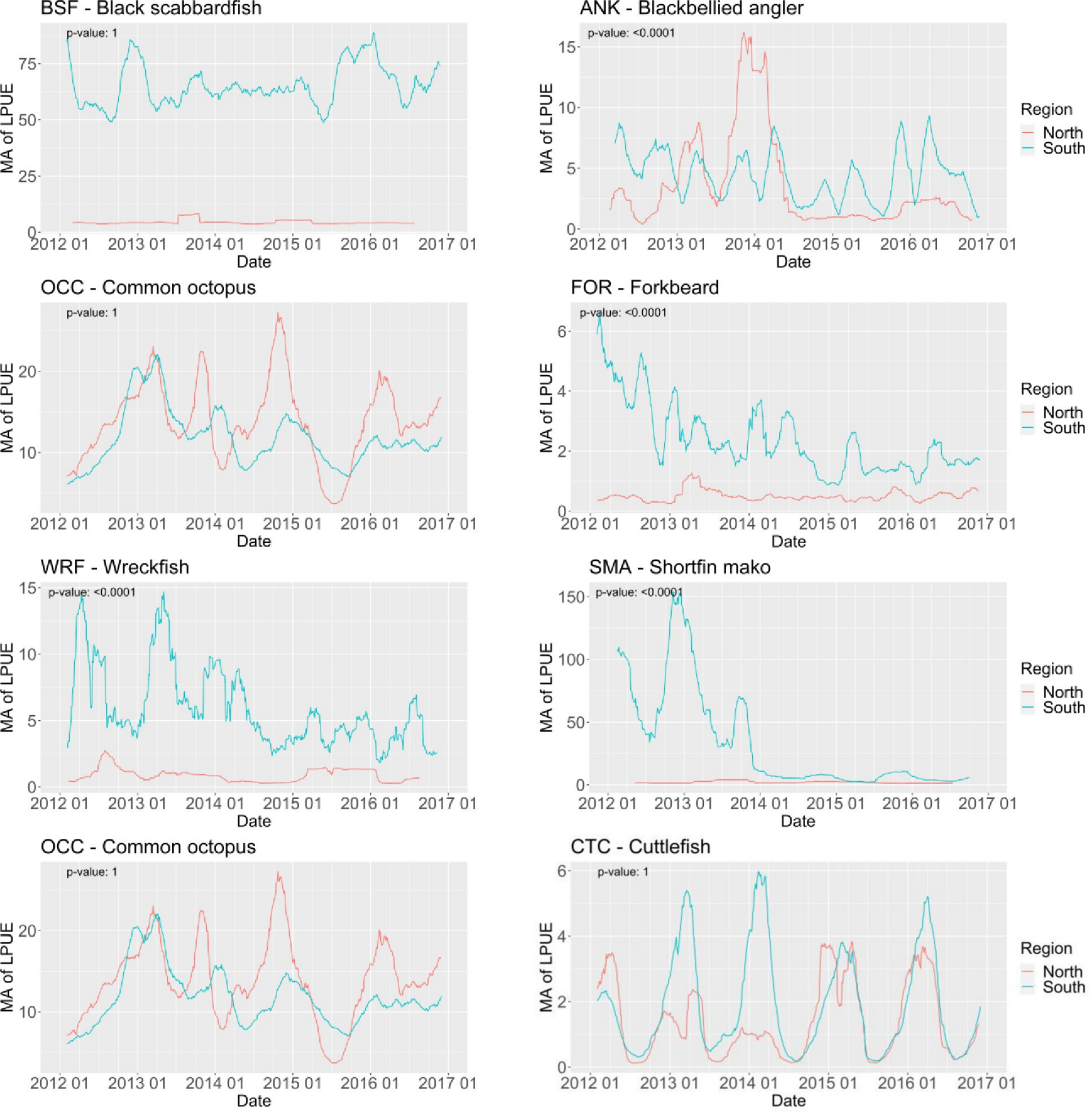
Daily landings per unit of effort (kg LoA-1 trip-1) expressed as moving average (MA) of 44 days (centre of classes plotted) for each species. The p-value of the linear trend is indicated at the top left corner of each plot. Blue and red lines correspond to the south and north regions, respectively. Species: a) black scabbardfish, b) blackbellied angler, c) common sole, d) forkbeard, e) wreckfish, f) shortfin mako, g) common octopus and h) cuttlefish.

The interpretation of the significance for the important explanatory variables used in the linear model (NAO index excluded) can be visualized in Fig 2. Year and season are compounded in the variable time (represented in the x axis) and region is taken into consideration by plotting for north and south separately. From the large number of species in analysis, only eight species were selected to be examined in detail. All these species are almost exclusively targeted by the multi-gear fleet, and none are subjected to formal assessment, being good candidates for the approach proposed in this work. Three are among the ten most important in quantity and/or value landed and are almost exclusively captured by the multi-gear fleet: black scabbardfish, common sole and common octopus. The blackbellied angler, forkbeard, the wreckfish and the cuttlefish are important species for specific segments of the multi-gear fleet. The shortfin mako is a threatened species with no formal assessment.

**Fig 3:**
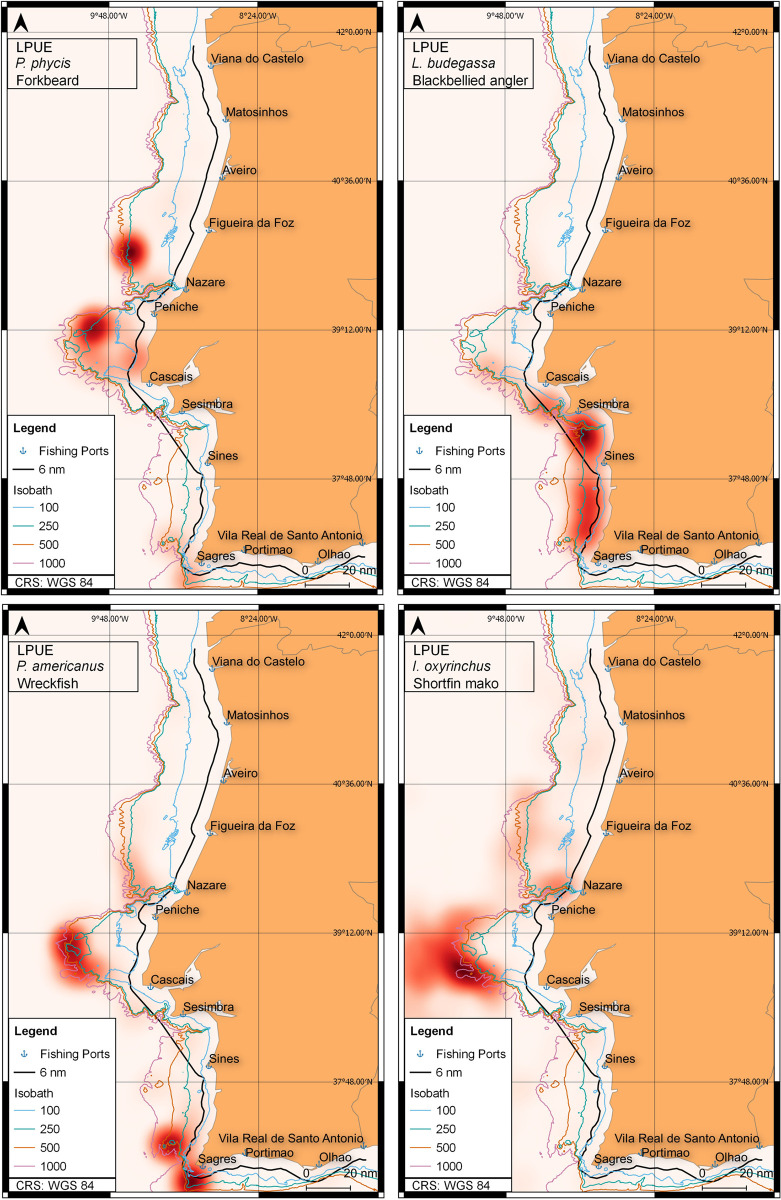
Heatmaps of LPUE (kg LoA-1 trip-1) used as a proxy for abundance, from 2012 to 2016, for a total of 4 species: a) forkbeard, b) blackbellied angler, c) wreckfish and d) shortfin mako. Main fishing ports are indicated as well as the 6 nautical miles line (inner limit of trawling activity). Bathymetry from the EMODnet Bathymetry Consortium [36].

Distribution maps of abundance (indicated by LPUE), based on landings and VMS fishing records, were produced for four of the previous species: blackbellied angler, forkbeard, shortfin mako and wreckfish (Fig 3).

The black scabbardfish is the second most important species landed. It is exclusively landed in the port of Sesimbra, being associated to the south area and despite significant higher average CPUE in the winter, it does not show clear seasonal fluctuations (Fig 2A). The blackbellied angler, with a significant reduction in LPUE over time (coef: -0.05; p-value < 0.0001), shows different trends in the north and south regions. The negative trend can be attributed to a significant drop in LPUE in the north after 2014 but, in the south, LPUE stayed stable (Fig 2B). For the south region, where seasonal fluctuations are clearer, there are two peaks in the spring and fall, this last one with higher average LPUE. The fishing effort for the blackbellied angler concentrates off Cascais and Peniche while higher LPUEs are located in the Sesimbra-Sagres coast, at fishing depths between 100 and 250 meters (Fig 3A). The common sole, with significant higher LPUE in the north (p-value < 0.0001), displays very similar patterns with respect to season in both areas (Fig 2C), likely due to migration patterns of the species. The forkbeard, landed mainly in the south, shows a significant decreasing trend with time as evidenced in Fig 3D (coef: -0.04; p-value < 0.0001). Superimposed on this trend, seasonal variations can be observed, with high average values in the spring but with peaks in early summer during most of the period in analysis. The LPUE suggests two hotspots, one North of the Nazaré canyon and a second between Cascais and Peniche (Fig 3B). Both areas are at 250m depth, close to the continental slope. The wreckfish also shows a decaying trend over time with a superimposed seasonal cycle, with higher LPUE in the summer (Fig 2E). It is also a species mostly captured in the south and is mainly concentrated in three hotspots, two off Sagres at around 250-500m depth and the third in the central region in the upper slope at depths around 500m depth (Fig 3C). A less dense area can be observed in the canyon off Nazaré.

The shortfin mako displays a very marked reduction from 2014 onwards (Fig 2F). This species, with the status “endangered” by the International Union for Conservation of Nature red list [38], was subject to a landing prohibition since 2021.

The two cephalopods, common octopus and cuttlefish, display typical seasonal variations related with their yearly lifecycles, with no yearly trends and higher catches in the fall and winter respectively. The cuttlefish has higher LPUE in the south, although in the last two years the values were similar in both regions, and it showed a significant correlation with the NAO index (coef: 0.07; p-value < 0.0001).

## Discussion

The assessment and management of the species caught by the multi-gear fleet analysed here is difficult due to the large number of species caught. A simple method that allows the temporal and spatial monitoring of these resources and provides information that can be used in management is very useful, as observed in other studies with similar objectives [39].

The index capture per unit of effort (CPUE) was one of the first indicators of abundance (biomass) of a resource used in stock assessment [40], under the assumption that CPUE is linearly correlated with stock biomass and may therefore be used as its indicator. The most common phenomena affecting the proportionality between CPUE and biomass, that may invalidate its use as an indicator of biomass is hyperstability [41], which is likely to occur when fishing in areas of high concentration of the species, for example spawning aggregations [42]. This situation may be combined with searching strategies targeting different concentrations of the species [43], causing CPUE to stay stable even when the resources decline. Other causes include abandonment of the fishery by less skilled fisherman when the stock declines [44] or increase in catchability due to gear improvement of better detection methods [45]. The stability of the fleet in the present study, with similar number of vessels and gears used, along with the short period analyzed, contributes to reducing the importance of the previously referred factors in the CPUE–abundance relationship.

Accurate estimation of the fishing effort was not possible, since finer measures of effort based on the gear characteristics (i.e., net length, number of hooks or traps or soaking time) were not available. Thus, vessel length was used to standardize fishing effort. Vessel length is an indicator that reflects the fishing capacity since there is an association between vessel’s length and the maximum fleet size (number of net panels or hooks) it is allowed to operate.

In this study CPUE was replaced by LPUE, assuming that catch and landings do not differ substantially due to low levels of discards. Of the different gears used by the multi-gear fleet the most important are longlines, tangling and trammel nets, and pots. Studies in discards are available for the Portuguese continental coast for trammel nets and pelagic longlines. For trammel nets there are several studies [24, 46–48] that indicate chub mackerel (*S*. *japonicus*), sardine (*Sardina pilchardus*) and longspine snipefish (*Macroramphosus scolopax*) as the most discarded species, followed by species occasionally discarded such as the longfin gurnard (*Chelidonichthys obscura*), dragonet (*Callioynimus lyra*) and bull ray (*Pteromylaeus bovines*). Regarding the pelagic longline [48], the most important species discarded were smooth lanternshark (*Etmopterus pusillus*), lesser-spotted dogfish (*Scyliorhinus canicula*), rays (*Raja spp*) and rabbit fish (*Chimaera monstrosa*). In any case, discards were almost entirely reported for species with no commercial value. Only two of these belong to the 48 selected species in this work, the chub mackerel and unspecified rays, invalidating the use of LPUE as an index of abundance in these cases. For all the other species, the assumption can be made that LPUE can replace CPUE as an indicator of abundance.

One last important issue related to the use of global indicators of abundance such as CPUE or LPUE is the assumption that catches are a random sample of the total range of distribution of the species, meaning no selection of specific fishing grounds or areas with high density [41]. Although not addressed in this study, the combination of LPUE with location (based on VMS data) can overcome this limitation allowing a spatial interpretation of the species distribution.

The reason for black scabbardfish, surf clam, swordfish, Atlantic pomfret and smooth clam having higher median values of LPUE (53, 27, 22, 21 and 20 kg LoA^-1^ trip^-1^ respectively) can be explained due to the intrinsic characteristics of the fisheries and the way the LPUE index was built. Besides fishing operations resulting in high catches, LPUE are favoured by longer trips or smaller vessels. The black scabbardfish (with median LPUE above 50) is targeted by a highly efficient deep bottom longline fishery with daily trips. The swordfish is caught by drifting longlines in trips that last for several days (typically three weeks) [49]. The Atlantic pomfret was reported as being by-caught by a longline fishery targeting hake [50] but no updated information on the possibility of this species being targeted is available, justifying specific studies. The two bivalve species (surf clam and smooth clam) have high median LPUE because they have individual daily quotas (in the order of hundreds of Kg) and the vessels have low LoA. The LPUE as expressed in this work is not a good indicator of the abundance of bivalve species due to the individual daily quota system.

The results of the linear models for LPUE as a function of year, NAO index, region and season, applied to the 48 most important species, showed a wide range of significant factors, indicating that a management strategy common to the whole fleet would be very difficult to apply. One clear output is the need to consider region when implementing management actions. In this study two regions were considered, north and south, separated by the Nazaré canyon on the west coast. While ubiquitous species such as the common octopus showed an even distribution throughout both regions, most species were strongly associated with the north or south areas, with southern species (lowfin gulper shark, shortfin mako, black scabbardfish, forkbeard, silver scabbardfish, swordfish and wreckfish) opposed to Northern ones (surf clam and neon flying squid).

Regarding the 41 species for which significant seasonal variation was estimated, all eight selected species for further graphical analysis showed higher LPUEs towards specific seasons. In some cases, abundance patterns of one species may be related to another species, as it is the case of the common sole and the cuttlefish which are mostly caught together. While the cuttlefish, after hatching in inshore waters migrates offshore (~6 nm) during the fall and winter [51], the common sole spawns in on-shore nurseries were the young stay during 2 years until moving with the adults to deeper waters during the same seasons [52]. Since the fleet’s fishing grounds for these species concentrate around the 200 meters depth, the maximum depth where both species are present, our findings suggest that higher availability to fisheries during the winter periods cause higher LPUE. The blackbellied angler also shows a distinctive seasonal pattern in LPUE with a steep decrease in the fall and further decrease in winter. This is attributed to the fishing closure of anglers (*Lophius* spp.) between January and February, corresponding to the reproduction period, to control the stock decline. For the Atlantic wreckfish and the shortfin mako LPUE was found to be significantly higher during the summer and fall respectively. Although caught in different seasons and with different fishing gears, both species are mainly targeted by the same, highly specific, group of vessels operating in the NE Atlantic, that often use seamounts as fishing grounds [49]. While the bottom longline is used during the summer periods to target wreckfish over the seamounts, the use of drifting longlines is more intense during the fall to mainly target swordfish, capturing also pelagic sharks such as shortfin mako [49]. Since wreckfish is a sedentary territorial species and shortfin mako a large pelagic migrator it is suggested that, although wreckfish and shortfin mako are caught all year, a highly specific fleet concentrates its effort during the summer and fall seasons. Due to this, when managing the wreckfish fisheries, the difference between the coastal and seamounts fleets should be considered. The black scabbardfish shows higher LPUE levels in the winter periods in comparison to other seasons. Although little is known about the life cycle of this deep water species, it is suggested it migrates from the West British Isles growing grounds to lower latitudes for reproduction purposes [53]. The higher LPUE associated to winter, which was also suggested in previous studies about this fleet [54], is yet to be explained. Finally, the forkbeard is mainly captured during spring, by the bottom longline fleet that targets more important commercial species such as blackbelly rosefish, blackspot seabream, red porgy and silver scabbardfish.

Significant trends over time (year) were observed for a total of 18 species, from which four displayed an increasing trend and the remaining a decreasing trend during the period in study. Although statistically significant, most of these trends have little practical impact since the absolute value of the slope, for log_10_(LPUE) as a function of year, is close to zero (for 14 species less than 0.1). The absence of clear trends is expected given the short period considered. For some species, landings were found to be restricted to some years, as it is the case of the sharks (lowfin gulper shark and shortfin mako), landed mostly from 2012 to 2014, with minor landings in subsequent years. For other species, such as neon flying squid and silver scabbardfish, peaks were registered in LPUE in 2016 and 2014–2015, respectively.

Only three species showed a significant correlation between the NAO index and LPUE: European hake, whiting and cuttlefish. European hake is not considered in the context of this work because it is mainly landed by coastal trawlers. With respect to cuttlefish, a non-significant relationship between the NAO index and the abundance of this species was reported for the Mediterranean Sea [55] but it is known that mollusc growth is influenced by temperature [56], and a positive correlation would be expected given the positive association between higher NAO indices and sea surface temperature [57]. With respect to whiting, a long-term study in the North Sea found no significant correlation between the species abundance and the NAO index [58] but in other studies, positive correlations were found. A correlation between the NAO index (warmer and dryer than average summer conditions) and the mean length of the age-0 group in the following winter was found for the population using the Bristol Channel as a nursery ground [59]. A positive corelation with abundance was also found for the population of the Thames estuary [60]. Better information of the spatial distribution of nursery grounds would be necessary to understand the possible relationship of the NAO index and whiting abundance off the Portuguese coast.

Species such as the common octopus, anglers, black scabbardfish, sole, swordfish and wreckfish are known to be targeted by this multi-gear fleet. Nevertheless, several species considered in this study are being captured as by-catch. It is the case of the gurnards (tub gurnard *C*. *lucerna*, *r*ed gurnard *A*. *cuculus and l*arge-scaled gurnard *L*. *cavillone*), wedge sole, *D*. *cuneata* and the Atlantic mackerel *S*. *japonicus*, captured in small nets with small mesh size. The same happens with skates (spotted ray, thornback ray, and blonde ray), caught in large mesh size nets.

Nine of the 48 species analysed are Chondrichthyes, known for their late-maturity and high longevity associated. Their high commercial interest makes them very vulnerable to overfishing [61], and two of them, the thornback ray and the blue shark rank 9 and 10 in importance due to amounts landed. The shortfin mako displayed a sharp decrease in 2014, which might have occurred as a result of a reduction in its capture or of unreporting / misreporting. According to ICCAT, this species was reported threatened in the North Atlantic during the period covered in this work due to bycatch of the swordfish fishing fleet [62]. Since 2019, the shortfin mako was introduced in the CITES appendix II [63] and in 2020 landings were forbidden in multiple European countries due to decreasing population.

Forkbeard, which is mainly caught as bycatch by the longline coastal fleet operating in the southern coast, showed a steep decrease. This is not the case in the north, where landings are lower and constant throughout the period in study. A population decline was previously considered in 2011–2012 when Atlantic and Mediterranean catches dropped by 50% [64]. The hypothesis of a stock decline starting in 2012 and continuing onward until, at least, 2016, the upper limit of the time range in our data, is plausible. Lastly, wreckfish, which is mainly caught by large longline vessels that operate in the Atlantic seamounts, also showed a decreasing trend during the 6-year period. This fleet shifts between drifting longline, for swordfish, and bottom longline, for demersal species such as wreckfish [49]. Current information regarding trends in wreckfish population abundance indicates a decrease since 2015, particularly in the Azores area [65]. The wreckfish is vulnerable to over-exploitation since it is long-lived, late maturity species, not subject to assessment or management measures. We hereby suggest that a monitoring program to evaluate their distribution (including age information) should be carried out.

Although modelling and mapping data are from two different sources, landings and VMS respectively, both are in line with the existence of regional differences in species assemblages. A previous study [27] demonstrated the existence of two fish assemblages separated by the Nazaré canyon, which is in accordance with the results obtained for the majority of the species in our study.

Trends in LPUE are a simple approach to characterize fisheries status in multi-species fisheries, where the high number of species involved makes the cost of fisheries independent stock assessment for all exploited species unrealistic. For many of these species, relative abundance based on landings or catch data is sometimes the only data available, as is the case for wreckfish, forkbeard, john dory and shortfin mako in this study. LPUE indexes provide the best available information for the implementation of management advice.

The approach used here needs to be expanded by improving the available information namely increasing the length of the time series analysed (to distinguish between random fluctuations and temporal trends) and considering information on migratory and lifecycle patterns (to correctly interpretate the geographical and seasonal changes in LPUE). In this work a simplistic approach with respect to region was considered, with only two zones included in the model but variations in fleet characteristics and oceanographic conditions, such as depth, may suggest a different partition of the area studied. The influence of the gear type on LPUE and the association of species caught and gear also needs to be considered, maybe complemented with interviews to the skippers to identify target and by-catch species.

In conclusion, it is suggested that the methodology proposed here, consisting in the analysis of LPUE in specific areas over time to indicate abundance, will be useful to monitor fished populations and to provide guidance for management when fisheries independent data are not available.

## Supporting information

S1 Table Summary table of North Atlantic Oscillation index time-lag (in years) of first recruitment to fisheries in for each species and respective reference. Minimum conservation reference size (MCRS) and previous studies were used to identify first recruitment to fisheries. Common name is sorted in alphabetic order inside each taxonomic class.
(DOCX)

S2 Table Importance of the landings of the 48 selected species (rank) by quantity (tones) and value (thousand Euros). Ten most important species in quantity landed and/or value landed are indicated in bold.
(DOCX)

S3 Table Indicators of LPUE (kg LoA-1 trip-1) distribution (minimum, 1st quartile, median, 3rd quartile and mean) for the 48 selected species. Species with median LPUE above 20 in bold.
(DOCX)
