# Correction: Time and spatial trends in landing per unit of effort as support to fisheries management in a multi-gear coastal fishery

**DOI:** 10.1371/journal.pone.0357978

**Published:** 2026-09-08

**Authors:** Pedro Leitão, Luis Sousa, Margarida Castro, Aida Campos

In Fig 2, the graph for SOL – Common Sole was inadvertently omitted due to a duplication error involving the OCC – Common Octopus graph. The authors have provided a corrected version of Fig 2 below.

**Fig 2:**
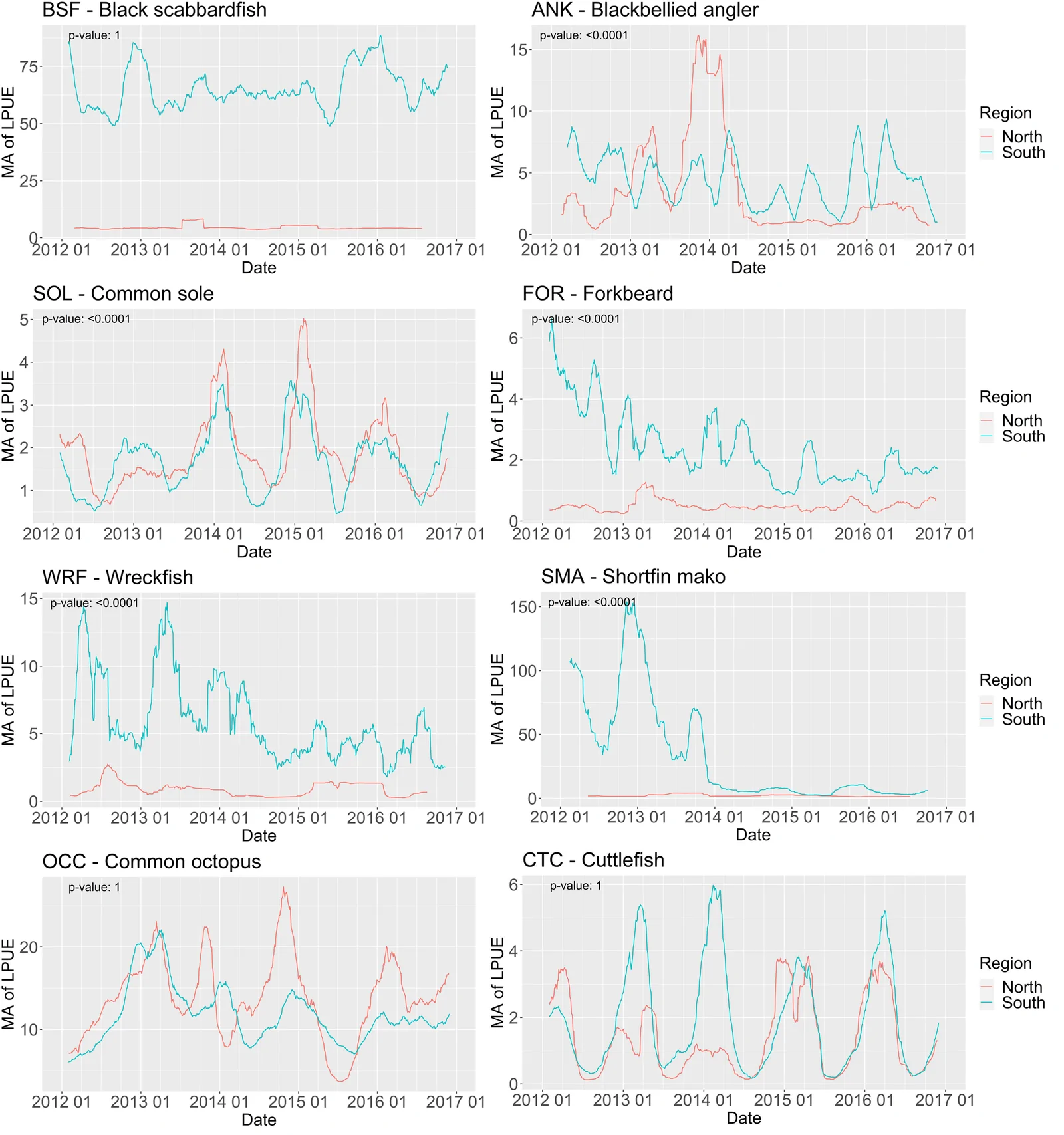
Daily landings per unit of effort (kg LoA-1 trip-1) expressed as moving average (MA) of 44 days (centre of classes plotted) for each species. The p-value of the linear trend is indicated at the top left corner of each plot. Blue and red lines correspond to the south and north regions, respectively. Species: a) black scabbardfish, b) blackbellied angler, c) common sole, d) forkbeard, e) wreckfish, f) shortfin mako, g) common octopus and h) cuttlefish.
