# Supplementary material for: Time and spatial trends in landing per unit of effort as support to fisheries management in a multi-gear coastal fishery
Source: PLoS One. 2022 Jul 1;17(7):e0258630. doi: 10.1371/journal.pone.0258630 (PMC9249400; doi:10.1371/journal.pone.0258630)
Supplement: S1 Table — Minimum conservation reference size (MCRS) and previous studies were used to identify first recruitment to fisheries. Common name is sorted in alphabetic order inside each taxonomic class. (DOCX) [file pone.0258630.s001.docx]

# SUPPLEMENTARY MATERIAL 1

| **Common name** | **NAO time-lag (years)** | **Reference** |
| --- | --- | --- |
| Bean clams | 1 | Growth and reproductive cycle of *Donax trunculus* L., (Mollusca: Bivalvia) off Faro, southern Portugal |
| Pod razor | 3 | Growth statistics of an exploited razor clam (*Ensis siliqua*) bed at Gormanstown, CoMeath, Ireland |
| Smooth clam | 6 | Age determination and growth rate of *Callista chione* population from the southwestern coast of Portugal |
| Stripped Venus clam | 2 | Age and growth of *chamelea gallina* from the algarve coast (southern portugal): influence of seawater temperature and gametogenic cycle on growth rate |
| Surf clam | 2 | Age and growth rate of the clam, *Spisula solida* L., from a site off Vilamoura, south Portugal, determined from acetate replicas of shell sections |
| Common octopus | 0 | Age and Growth of *Octopus vulgaris* Cuvier, 1797, Along the East Coast of Tunisia |
| Cuttlefish | 0 | Trends in age-at-recruitment and juvenile growth of cuttlefish, *Sepia officinalis*, from the English Channel |
| Neon flying squid | 0 | Age and growth of the neon flying squid, *Ommastrephes bartrami*, in the North Pacific Ocean |
| Blonde ray | 4 | Age, growth, sexual maturity and reproduction of the thornback ray, *Raja clavata* (L.), of the Gulf of Gabès (south-central Mediterranean Sea) |
| Blue shark | 4 | Age and growth of the blue shark (*Prionace glauca*) in the North Atlantic Ocean |
| Lowfin gulper shark | 21 | Age estimation of the exploited deepwater shark *Centrophorus squamosus* from the continental slopes of theRockall Trough and Porcupine Bank |
| Nursehound | 0 | Age Determination, Growth and Reproduction in the Lesser-spotted Dogfish, Scyliorhinus canicula (L.) |
| Shortfin mako | 0 | Age, Growth and Spatial Distribution of the Life Stages of the Shortfin Mako, Isurus oxyrinchus (Rafinesque, 1810) Caught in the Western and Central Atlantic |
| Smooth-hound | 0 | Preliminary study on age, growth and reproduction of Mustelus mustelus (Elasmobranchii: Carcharhiniformes: Triakidae) inhabiting the Gulf of Iskenderun, north-eastern Mediterranean Sea |
| Spotted ray | 3 | Age, Growth and Maturity of the Commercial Ray Species from the Irish Sea |
| Thornback ray | 5 | Age and growth of Raja clavata Linnaeus, 1758 – evaluation of ageing precision using different types of caudal denticles |
| Tope Shark | 2 | Age and growth of New Zealand school shark, Galeorhinus galeus |
| Angler | 0 | Age and growth of anglerfish (Lophius piscatorius) on the Porcupine Bank (west of Ireland) based on illicia age estimation |
| Atlantic horse mackerel | 0 | Growth and reproduction of horse mackerel, Trachurus trachurus (carangidae) |
| Atlantic mackerel | 0 | Age and growth of northeast atlantic mackerel (scomber scombrus) in waters of the north and northwest of spain (ices divisions viiic and ixa north), 1990-2000 |
| Atlantic pomfret | 4 | Age and growth of Ray's bream (Brama brama) from the south of Portugal |
| Axillary seabream | 2 | Age, growth and reproduction of the axillary seabream, Pagellus acarne (Risso, 1827), from the South coast of Portugal |
| Black scabbardfish | 3 | Age and growth of the black scabbard fish (Aphanopus carbo) off Madeira |
| Black seabream | 1 | Age and growth of Spondyliosoma cantharus (Sparidae) in the Gulf of Tunis |
| Blackbellied angler | 1 | On the biology and growth of the anglerfish Lophius budegassa Spinola, 1807 in the Spanish Mediterranean: a preliminary approach |
| Blackbelly rosefish | 8 | Age and growth of bluemouth, Helicolenus dactylopterus, from the Portuguese continental slope |
| Blackspot seabream | 4 | Studies on Age Determination and Growth Pattern of the Red (Blackspot) Seabream [Pagellus bogaraveo (Brünnich, 1768)] from the Strait of Gibraltar (ICES IXa/SW Spain): Application of the Species Migratory Pattern |
| Chub mackerel | 1 | Age and growth of the Atlantic chub mackerel Scomber colias Gmelin, 1789 off Madeira Island |
| Common sole | 2 | Comparative analysis of the diet, growth and reproduction of the soles, Solea solea and Solea senegalensis, occurring in sympatry along the Portuguese coast |
| Common two-banded seabream | 2 | Age and growth, maturity, mortality and yield-per-recruit for two banded bream (Diplodus vulgaris Geoffr.) from the south coast of Portugal |
| European conger | 2 | Age, growth and reproductive biology of the European conger eel (Conger conger) from the Atlantic Iberian waters |
| European hake | 2 | Age estimation, growth and maturity of the European hake (Merluccius merluccius (Linnaeus, 1758)) from Iberian Atlantic waters |
| European seabass | 5 | Movements, abundance, age composition and growth of bass, dicentrarchus labrax, in the severn estuary and inner bristol channel |
| Forkbeard | 2 | Age and growth of the forkbeard Phycis phycis (Gadidae) from the Azorean archipelago, North Atlantic |
| John dory | 1 | Age, growth, reproduction and feeding of John Dory, Zeus faber (Pisces: Zeidae), in the Saros Bay (North Aegean Sea) |
| Large-scaled gurnard | 0 | Growth and reproduction of large-scaled gurnard (Lepidotrigla cavillone Lacepède, 1801) (Triglidae) in the central Aegean Sea, eastern Mediterranean |
| Meagre | 2 | Marking of otoliths, age validation and growth of Argyrosomus regius juveniles (Sciaenidae) |
| Pouting | 2 | Age and growth of Trisopterus luscus (Linnaeus, 1758) (Pisces, Gadidae) off the coast of Asturias |
| Red gurnard | 0 | Population biology of the red gurnard (Aspitrigla cuculus L.; Triglidae) in the inshore waters of Eastern Anglesey and Northwest Wales |
| Red porgy | 2 | Age, growth and mortality of the red porgy, pagrus pagrus, in the eastern mediterranean sea (dodecanese, greece) |
| Sand sole | 2 | Feeding ecology, growth and sexual cycle of the sand sole, Solea lascaris, along the Portuguese coast |
| Silver scabbardfish | 4 | Using information-based methods to model age and growth of the silver scabbardfish, Lepidopus caudatus , from the mid-Atlantic Ocean |
| Surmullet | 1 | Croissance du rouget de roche Mullus surmuletus dans le nord du golfe de Gascogne |
| Swordfish | 2 | Age determination and growth of swordfish Xiphias gladius L., 1758 in the Aegean Sea |
| Tub gurnard | 0 | Age, growth and maturity of tub gurnard (Chelidonichthys lucerna Linnaeus 1758; Triglidae) in the inshore coastal waters of Northwest Wales, UK |
| Wedge sole | 1 | Studies on age determination and growth pattern of the wedge sole Dicologoglossa cuneata (Moreau, 1881) in the Spanish waters of the Gulf of Cadiz (southwest Iberian Peninsula) |
| Whiting | 7 | Determination of Some Population Parameters and Reproduction Period of Whiting (Merlangius merlangus euxinus Nordmann, 1840) on the Trabzon-Yomra Coast in the Eastern Black Sea |
| Wreckfish | 1 | Age and growth of southwestern Atlantic wreckfish Polyprion americanus |
