# Supplementary material for: Time and spatial trends in landing per unit of effort as support to fisheries management in a multi-gear coastal fishery
Source: PLoS One. 2022 Jul 1;17(7):e0258630. doi: 10.1371/journal.pone.0258630 (PMC9249400; doi:10.1371/journal.pone.0258630)
Supplement: S2 Table — Ten most important species in quantity landed and/or value landed are indicated in bold. (DOCX) [file pone.0258630.s002.docx]

# SUPPLEMENTARY MATERIAL 2

| **Common name** | **Scientific name** | **FAO code** | Landings in weight | | | Landings value | | |
| --- | --- | --- | --- | --- | --- | --- | --- | --- |
|  |  |  | rank | tones | % total | rank | thousand € | % total |
| *Actinopterygii* |  |  |  |  |  |  |  |  |
| **Angler** | ***Lophius piscatorius*** | **MON** | 11 | 1 365.7 | 1.78 | **9** | **6 823** | 2.63 |
| **Atlantic horse mackerel** | ***Trachurus trachurus*** | **HOM** | **4** | **4 472.8** | **5.82** | 12 | 5 187 | 2.0 |
| Atlantic mackerel | *Scomber scombrus* | MAC | 32 | 251.5 | 0.3 | 46 | 216 | 0.1 |
| Atlantic pomfret | *Brama brama* | POA | 25 | 466.1 | 0.6 | 37 | 825 | 0.3 |
| Axillary seabream | *Pagellus acarne* | SBA | 26 | 448.2 | 0.6 | 24 | 1 852 | 0.7 |
| **Black scabbardfish** | ***Aphanopus carbo*** | **BSF** | **2** | **9 460.8** | **12.30** | **2** | **27 173** | **10.46** |
| Black seabream | *Spondyliosoma cantharus* | BRB | 39 | 178.6 | 0.2 | 41 | 437 | 0.2 |
| Blackbellied angler | *Lophius budegassa* | ANK | 22 | 580.9 | 0.8 | 17 | 3 382 | 1.3 |
| Blackbelly rosefish | *Helicolenus dactylopterus* | BRF | 28 | 427.6 | 0.6 | 25 | 1 709 | 0.7 |
| Blackspot seabream | *Pagellus bogaraveo* | SBR | 35 | 200.5 | 0.3 | 18 | 2 633 | 1.0 |
| Chub mackerel | *Scomber japonicus* | MAS | 27 | 430.0 | 0.6 | 44 | 295 | 0.1 |
| **Common sole** | ***Solea solea*** | **SOL** | 12 | 1 301.8 | 1.7 | **5** | **11 651** | 4.5 |
| Common two-banded seabream | *Diplodus vulgaris* | CTB | 45 | 140.0 | 0.2 | 42 | 379 | 0.1 |
| **European conger** | ***Conger conger*** | **COE** | **7** | **2 400.4** | **3.12** | **10** | **6 106** | **2.35** |
| **European hake** | ***Merluccius merluccius*** | **HKE** | **3** | **6 709.5** | **8.72** | **3** | **19 264** | **7.42** |
| European seabass | *Dicentrarchus labrax* | BSS | 24 | 486.5 | 0.6 | 14 | 4 159 | 1.6 |
| Forkbeard | *Phycis phycis* | **FOR** | 18 | 700.6 | 0.9 | 19 | 2 199 | 0.8 |
| John dory | *Zeus faber* | JOD | 13 | 972.3 | 1.3 | **7** | **9 014** | 3.5 |
| Large-scaled gurnard | *Lepidotrigla cavillone* | LDV | 23 | 528.6 | 0.7 | 29 | 1 234 | 0.5 |
| Meagre | *Argyrosomus regius* | MGR | 47 | 131.9 | 0.2 | 33 | 905 | 0.3 |
| **Pouting** | ***Trisopterus luscus*** | **BIB** | **5** | **4 345.6** | **5.65** | **8** | **7 253** | **2.79** |
| Red gurnard | *Aspitrigla cuculus* | GUR | 44 | 144.7 | 0.2 | 48 | 145 | 0.1 |
| Red porgy | *Pagrus pagrus* | RPG | 40 | 169.5 | 0.2 | 20 | 2 183 | 0.8 |
| Sand sole | *Pegusa lascaris* | SOS | 34 | 248.0 | 0.3 | 28 | 1 427 | 0.5 |
| Silver scabbardfish | *Lepidopus caudatus* | SFS | 38 | 182.5 | 0.2 | 30 | 1 100 | 0.4 |
| Surmullet | *Mullus surmuletus* | MUR | 36 | 189.5 | 0.2 | 21 | 2 133 | 0.8 |
| **Swordfish** | ***Xiphias gladius*** | **SWO** | **8** | **2 338.6** | **3.04** | **4** | **13 665** | **5.26** |
| Tub gurnard | *Chelidonichthys lucerna* | GUU | 43 | 166.2 | 0.2 | 43 | 352 | 0.1 |
| Wedge sole | *Dicologlossa cuneata* | CET | 30 | 379.0 | 0.5 | 26 | 1 555 | 0.6 |
| Whiting | *Merlangius merlangus* | WHG | 41 | 168.7 | 0.2 | 35 | 867 | 0.3 |
| Wreckfish | *Polyprion americanus* | WRF | 31 | 360.5 | 0.5 | 11 | 5 918 | 2.3 |
| *Chondrichthyes* |  |  |  |  |  |  |  |  |
| Blonde ray | *Raja brachyura* | RJH | 19 | 689.7 | 0.9 | 23 | 1 912 | 0.7 |
| **Blue shark** | ***Prionace glauca*** | **BSH** | **10** | **1 549.1** | **2.01** | 22 | 2 012 | 0.8 |
| Lowfin gulper shark | *Centrophorus lusitanicus* | CPL | 16 | 843.6 | 1.1 | 27 | 1 428 | 0.5 |
| Nursehound | *Scyliorhinus stellaris* | SYT | 21 | 642.4 | 0.8 | 39 | 446 | 0.2 |
| Shortfin mako | *Isurus oxyrinchus* | SMA | 15 | 958.7 | 1.2 | 13 | 4 321 | 1.7 |
| Smooth-hound | *Mustelus mustelus* | SMD | 33 | 251.3 | 0.3 | 34 | 892 | 0.3 |
| Spotted ray | *Raja montagui* | RJM | 42 | 167.0 | 0.2 | 40 | 440 | 0.2 |
| **Thornback ray** | ***Raja clavata*** | **RJC** | **9** | **1 659.7** | **2.16** | 15 | 4 148 | 1.6 |
| Tope Shark | *Galeorhinus galeus* | GAG | 48 | 110.5 | 0.1 | 47 | 199 | 0.1 |
| Cephalopoda |  |  |  |  |  |  |  |  |
| **Common octopus** | ***Octopus vulgaris*** | **OCC** | **1** | **22 252.0** | **28.93** | **1** | **84 534** | **32.56** |
| Cuttlefish | *Sepia officinalis* | CTC | 14 | 961.1 | 1.2 | 16 | 3 776 | 1.5 |
| Neon flying squid | *Ommastrephes bartramii* | OFJ | 46 | 137.4 | 0.2 | 45 | 246 | 0.1 |
| Bivalves |  |  |  |  |  |  |  |  |
| Bean clams | *Donax spp* | DON | 29 | 381.6 | 0.5 | 31 | 1 024 | 0.4 |
| Pod razor | *Ensis siliqua* | EQI | 37 | 188.1 | 0.2 | 38 | 556 | 0.2 |
| Smooth clam | *Callista chione* | KLK | 17 | 840.2 | 1.1 | 36 | 862 | 0.3 |
| Stripped Venus clam | *Chamelea gallina* | SVE | 20 | 675.6 | 0.9 | 32 | 1 014 | 0.4 |
| **Surf clam** | ***Spisula solida*** | **ULO** | **6** | **4 250.7** | **5.53** | **6** | **9 813** | **3.78** |
