# Supplementary material for: Time and spatial trends in landing per unit of effort as support to fisheries management in a multi-gear coastal fishery
Source: PLoS One. 2022 Jul 1;17(7):e0258630. doi: 10.1371/journal.pone.0258630 (PMC9249400; doi:10.1371/journal.pone.0258630)
Supplement: S3 Table — Species with median LPUE above 20 in bold. (DOCX) [file pone.0258630.s003.docx]

# SUPPLEMENTARY MATERIAL 3

| **Common name** | **Scientific name** | **FAO code** | **Min** | **1st Q** | **Median** | **3rd Q** | **Max** | **Mean** |
| --- | --- | --- | --- | --- | --- | --- | --- | --- |
| *Actinopterygii* |  |  |  |  |  |  |  |  |
| Angler | *Lophius piscatorius* | MON | 0.1 | 1.0 | 2.7 | 6.0 | 59.8 | 4.2 |
| Atlantic horse mackerel | *Trachurus trachurus* | HOM | 0.1 | 2.2 | 6.1 | 12.2 | 309.6 | 9.7 |
| Atlantic mackerel | *Scomber scombrus* | MAC | 0.1 | 0.4 | 0.7 | 1.7 | 51.8 | 1.9 |
| **Atlantic pomfret** | ***Brama brama*** | **POA** | **0.0** | **0.6** | **21.2** | **90.9** | **715.7** | **61.2** |
| Axillary seabream | *Pagellus acarne* | SBA | 0.0 | 0.4 | 0.6 | 1.0 | 11.4 | 0.8 |
| **Black scabbardfish** | ***Aphanopus carbo*** | **BSF** | **0.1** | **4.6** | **53.5** | **70.9** | **187.3** | **45.2** |
| Black seabream | *Spondyliosoma cantharus* | BRB | 0.0 | 0.2 | 0.3 | 0.5 | 83.1 | 0.8 |
| Blackbellied angler | *Lophius budegassa* | ANK | 0.0 | 0.6 | 1.8 | 5.0 | 110.4 | 3.9 |
| Blackbelly rosefish | *Helicolenus dactylopterus* | BRF | 0.0 | 0.5 | 1.0 | 2.2 | 111.6 | 2.6 |
| Blackspot seabream | *Pagellus bogaraveo* | SBR | 0.0 | 0.2 | 0.6 | 2.1 | 94.9 | 2.2 |
| Chub mackerel | *Scomber japonicus* | MAS | 0.0 | 0.7 | 1.5 | 3.2 | 710.9 | 5.9 |
| Common sole | *Solea solea* | SOL | 0.0 | 0.8 | 1.4 | 2.1 | 37.4 | 1.8 |
| Common two-banded seabream | *Diplodus vulgaris* | CTB | 0.0 | 0.2 | 0.3 | 0.5 | 42.9 | 0.5 |
| European conger | *Conger conger* | COE | 0.1 | 1.6 | 3.1 | 5.9 | 228.3 | 6.5 |
| European hake | *Merluccius merluccius* | HKE | 0.1 | 3.6 | 6.3 | 10.4 | 167.3 | 8.2 |
| European seabass | *Dicentrarchus labrax* | BSS | 0.0 | 0.2 | 0.6 | 1.8 | 382.3 | 2.1 |
| Forkbeard | *Phycis phycis* | FOR | 0.0 | 0.3 | 0.8 | 1.6 | 37.3 | 1.5 |
| John dory | *Zeus faber* | JOD | 0.0 | 0.7 | 1.8 | 3.7 | 36.3 | 2.8 |
| Large-scaled gurnard | *Lepidotrigla cavillone* | LDV | 0.0 | 0.3 | 0.4 | 0.7 | 89.4 | 0.9 |
| Meagre | *Argyrosomus regius* | MGR | 0.0 | 0.3 | 0.8 | 1.8 | 53.8 | 1.8 |
| Pouting | *Trisopterus luscus* | BIB | 0.1 | 1.0 | 1.8 | 4.6 | 32.4 | 3.3 |
| Red gurnard | *Aspitrigla cuculus* | GUR | 0.0 | 0.3 | 0.4 | 0.6 | 13.2 | 0.6 |
| Red porgy | *Pagrus pagrus* | RPG | 0.0 | 0.2 | 0.4 | 0.9 | 31.8 | 1.0 |
| Sand sole | *Pegusa lascaris* | SOS | 0.0 | 0.2 | 0.4 | 0.7 | 14.8 | 0.7 |
| Silver scabbardfish | *Lepidopus caudatus* | SFS | 0.1 | 0.6 | 2.5 | 13.0 | 95.5 | 10.5 |
| Surmullet | *Mullus surmuletus* | MUR | 0.0 | 0.2 | 0.3 | 0.5 | 6.4 | 0.4 |
| **Swordfish** | ***Xiphias gladius*** | **SWO** | **0.4** | **3.9** | **21.9** | **179.6** | **805.3** | **105.8** |
| Tub gurnard | *Chelidonichthys lucerna* | GUU | 0.0 | 0.3 | 0.5 | 0.8 | 10.1 | 0.7 |
| Wedge sole | *Dicologlossa cuneata* | CET | 0.0 | 0.2 | 0.5 | 3.0 | 44.3 | 2.9 |
| Whiting | *Merlangius merlangus* | WHG | 0.0 | 0.3 | 0.6 | 1.3 | 51.0 | 1.4 |
| Wreckfish | *Polyprion americanus* | WRF | 0.1 | 0.4 | 1.1 | 3.4 | 155.4 | 4.2 |
| *Chondrichthyes* |  |  |  |  |  |  |  |  |
| Blonde ray | *Raja brachyura* | RJH | 0.1 | 1.0 | 1.9 | 3.5 | 38.1 | 2.9 |
| Blue shark | *Prionace glauca* | BSH | 0.1 | 0.6 | 1.2 | 2.9 | 815.1 | 23.6 |
| Lowfin gulper shark | *Centrophorus lusitanicus* | CPL | 0.3 | 10.4 | 14.9 | 22.4 | 162.5 | 22.2 |
| Nursehound | *Scyliorhinus stellaris* | SYT | 0.1 | 1.0 | 1.4 | 2.0 | 30.2 | 1.7 |
| Shortfin mako | *Isurus oxyrinchus* | SMA | 0.1 | 1.0 | 2.5 | 12.0 | 595.2 | 28.2 |
| Smooth-hound | *Mustelus mustelus* | SMD | 0.0 | 0.4 | 0.6 | 1.0 | 243.1 | 6.1 |
| Spotted ray | *Raja montagui* | RJM | 0.1 | 0.5 | 0.9 | 1.6 | 37.9 | 1.5 |
| Thornback ray | *Raja clavata* | RJC | 0.1 | 1.1 | 1.8 | 2.8 | 60.9 | 2.3 |
| Tope Shark | *Galeorhinus galeus* | GAG | 0.0 | 3.8 | 5.0 | 5.9 | 18.7 | 4.9 |
| *Cephalopoda* |  |  |  |  |  |  |  |  |
| Common octopus | *Octopus vulgaris* | OCC | 0.1 | 8.4 | 11.6 | 15.5 | 93.2 | 12.8 |
| Cuttlefish | *Sepia officinalis* | CTC | 0.0 | 0.3 | 0.9 | 2.2 | 38.2 | 1.7 |
| Neon flying squid | *Ommastrephes bartramii* | OFJ | 0.0 | 0.3 | 0.7 | 1.3 | 86.7 | 1.3 |
| *Bivalves* |  |  |  |  |  |  |  |  |
| Bean clams | *Donax spp* | DON | 0.8 | 5.4 | 6.9 | 8.5 | 37.9 | 7.2 |
| Pod razor | *Ensis siliqua* | EQI | 1.8 | 7.7 | 10.9 | 15.5 | 62.1 | 12.6 |
| **Smooth clam** | ***Callista chione*** | **KLK** | **0.8** | **15.7** | **20.2** | **25.6** | **68.2** | **21.2** |
| Stripped Venus clam | *Chamelea gallina* | SVE | 0.5 | 6.2 | 9.5 | 12.9 | 58.9 | 9.9 |
| **Surf clam** | ***Spisula solida*** | **ULO** | **1.3** | **10.7** | **27.5** | **54.9** | **147.4** | **34.1** |
